# Development of a Rapid Reverse Transcription-Recombinase Polymerase Amplification Couple Nucleic Acid Lateral Flow Method for Detecting Porcine Epidemic Diarrhoea Virus

**DOI:** 10.3390/biology11071018

**Published:** 2022-07-06

**Authors:** Seatthanan Pewlaoo, Siratcha Phanthong, Thida Kong-Ngoen, Sirijan Santajit, Witawat Tunyong, Shutipen Buranasinsup, Kampon Kaeoket, Techit Thavorasak, Pornpan Pumirat, Nitat Sookrung, Wanpen Chaicumpa, Nitaya Indrawattana

**Affiliations:** 1 Department of Microbiology and Immunology, Faculty of Tropical Medicine, Mahidol University, Bangkok 10400, Thailand; seatthanan.pew@gmail.com (S.P.); thida.kon@mahidol.ac.th (T.K.-N.); witawat.tun@mahidol.ac.th (W.T.); pornpan.pum@mahidol.ac.th (P.P.); 2 Siriraj Center of Research Excellence in Allergy and Immunology, Faculty of Medicine Siriraj Hospital, Mahidol University, Bangkok 10700, Thailand; siratcha.pha@mahidol.ac.th (S.P.); nitat.soo@mahidol.ac.th (N.S.); 3 Center of Research Excellence on Therapeutic Proteins and Antibody Engineering, Department of Parasitology, Faculty of Medicine Siriraj Hospital, Mahidol University, Bangkok 10700, Thailand; techit.tha@student.mahidol.ac.th (T.T.); wanpen.cha@mahidol.ac.th (W.C.); 4 Department of Medical Technology, School of Allied Health Sciences, Walailak University, Nakhon Si Thammarat 80160, Thailand; sirijan.sa@wu.ac.th; 5 Research Center in Tropical Pathobiology, Walailak University, Nakhon Si Thammarat 80160, Thailand; 6 Department of Pre-Clinic and Applied Animal Science, Faculty of Veterinary Science, Mahidol University, Nakhon Pathom 73170, Thailand; shutipen.bur@mahidol.ac.th; 7 Department of Clinical Sciences and Public Health, Faculty of Veterinary Science, Mahidol University, Nakhon Pathom 73170, Thailand; kampon.kae@mahidol.ac.th; 8 Biomedical Research Incubator Unit, Department of Research, Faculty of Medicine Siriraj Hospital, Mahidol University, Bangkok 10700, Thailand

**Keywords:** porcine epidemic diarrhea virus, RT-qPCR, RT-RPA-NALF, rapid detection

## Abstract

**Simple Summary:**

Porcine epidemic diarrhea virus infection is an important acute diarrheal disease of swine especially in infected piglets can caused severe diarrhea, dehydration with difficulty in digesting milk curd, leading to death. The diagnosis of this viral infection is essential for monitoring and managing the disease. There is surprisingly little evidence such as easy rapid detection in the field. In this study, we developed rapid the reverse transcription-recombinase polymerase amplification couple nucleic acid lateral flow for Porcine epidemic diarrhea virus detection targeted the membrane gene in the genome sequence of the virus. Herein, the results shown that the established assay is simple and rapid, increases high sensitivity and specificity, and can be applied in the field.

**Abstract:**

Porcine epidemic diarrhea virus (PEDV) infection is an important acute diarrheal disease of swine that results in economic and industrial losses worldwide. The clinical manifestations in infected piglets are severe diarrhea, dehydration with milk curd indigestion, leading to death. The diagnosis of PEDV is essential for monitoring and managing the disease. PEDV can be detected and identified by serology and the nucleic acid of the virus in clinical samples. Therefore, a novel isothermal amplification and detection technique, reverse transcription-recombinase polymerase amplification couple nucleic acid lateral flow (RT-RPA-NALF) was developed for the rapid detection of PEDV. Qualitative reverse transcription-polymerase chain reaction (RT-qPCR) was established as the gold standard assay to compare results. Specific primer pairs and probes were designed, and RT-RPA conditions were optimized to amplify the M gene of PEDV. The established RT-RPA-NALF assay could finish in 25 min at a temperature of 42 °C and the amplicon interpreted by visual detection. The developed RT-RPA-NALF assay was specific to the M gene of PEDV, did not detect other common swine diarrhea pathogens, and showed minimal detection at 10^2^ TCID_50_/mL PEDV. The RT-RPA-NALF assay can detect PEDV in 5 simulated fecal samples. Furthermore, in 60 clinical fecal samples, the results of RT-RPA-NALF correlated with RT-qPCR assay, which provides sensitivity of 95.65% and specificity of 100%, with a coincident rate of 98.33%. The rapid RT-RPA-NALF is simple and rapid, increases high sensitivity, and can be used in the field.

## 1. Introduction

Porcine epidemic diarrhoea (PED) is an acute diarrhoeal disease in swine caused by the porcine epidemic diarrhoea virus (PEDV), which results globally in economic and industrial losses among swine [1,2,3]. PEDV transmission occurs through direct and indirect fecal-oral contact with infected waste, contaminated tools, environmental sources, such as people, wild animals [2], or airborne transmission [4]. PEDV infects the oral route and replicates in villous epithelial cells of the small intestine, particularly the jejunum and ileum, which reduces the villi length and destroys the cells, leading to apoptosis [1,4]. Clinical manifestations of infected swine of all ages include severe diarrhoea and dehydration with milk curd indigestion, leading to death. However, the severity of the disease depends on the age of the infected swine. In particular, piglets have high morbidity and mortality rates [4].

PEDV, which belongs to the family *Coronaviridae*, genus *Alphacoronavirus* and order *Nidovirales,* is an enveloped virus with single-strand positive RNA in approximately 28-kb encoded non-structural and structural proteins [3,5]. The non-structural protein (nsp) is cleaved into 16 nsp. The structural proteins comprise spike (S) protein, accessory proteins (ORF3), envelope (E) protein, membrane (M) protein and nucleocapsid (N) [3]. PEDV can be divided into genogroups 1 (G1) and 2 (G2) [5]. Porcine endemic diarrhoea emerged in the early 1970s in Belgium and England, and the causative virus was identified as a PEDV CV777 prototype [6]. Then, it spread to the other parts of Europe, such as the Netherlands and Hungary [4,6]. Furthermore, PEDV emerged and has been isolated in Asia since the 1980s [4,6,7] In Thailand, a country in Asia, PEDV was found and emerged first in 2007 and was identified as genotype 2 and as both genotypes later [5,8]. In addition, it has been reported that the variation of PEDV in Thailand is related to Chinese and Korean isolates [9].

PEDV diagnosis is possible and essential for monitoring and managing the disease. From clinical manifestations, lesion and histopathology of the intestine, which was observed in the gastrointestinal tract by the intestine having a thin wall and much accumulated yellow fluid, the stomach filled with curdled milk [7], are not appropriate detection methods [10]. However, in clinical samples, it can be detected and identified by serology and the nucleic acids of the virus [11]. In serological detection, including virus isolation, enzyme-linked immunosorbent assay is laborious and time-consuming. Therefore, nucleic acid diagnosis, such as PCR and real-time RT-PCR (RT-qPCR), is preferred, because it is quick, convenient, specific and sensitive [10,11,12]. Isothermal amplification is an advanced nucleic acid amplification method that uses temperature to amplify the target DNA or RNA, such as loop-mediated isothermal amplification (LAMP), recombinase polymerase amplification (RPA) and helicase amplification [13].

RPA is a nucleotide chain amplification technique through a single-temperature (isothermal) reaction that can be used instead of a polymerase chain reaction (PCR). The RPA technique offers the advantages of simplicity, speed, accuracy and low cost compared with conventional PCR; it is quick and uses fewer primers than the LAMP technique [14]. The principle of the RPA technique is that three main enzymes are used: recombinase enzyme, single-stranded DNA binding protein (SSB) and strand-displacing polymerase. The reaction begins with the recombinase enzyme pairing the oligonucleotide primer with the homologous sequence in the DNA duplex [14]; SSB inserts the replaced DNA strand, forming a D loop. The strand-displacing polymerase then initiates DNA synthesis, where the specific forward and reverse primers bind to the DNA target. The exponential DNA amplification reaction is then initiated. This technique does not require additional heat to split the DNA strand or reduced heat to allow the nucleotide to pair. RPA reactions are performed at temperatures of 37 °C–42 °C, where the reaction proceeds rapidly and results in the amplification of specific DNA from a few copies of the target DNA to detectable levels, usually in a short turnaround time of around 30 min. RPA techniques have been developed for widespread diagnostic use, such as rapid DNA or RNA detection of the genome of the Rift Valley fever virus [15], bovine coronavirus [16], foot-and-mouth disease virus [17] and HIV-1 proviral DNA [18], as well as the genomic DNA detection of pathogenic bacteria, such as *Francisella tularensis* [19], biothreat agents [20], *Neisseria gonorrhoeae*, *Salmonella enterica*, methicillin-resistant *Staphylococcus aureus* (MRSA) [21] and *Cronobacter* spp. [22].

Recently, a method to read target gene proliferation results has been developed by adding a fluorophore-labelled probe (quencher) to the reaction. This allows real-time test results to be reported, or the reaction results can be read in the nucleic acid lateral flow (NALF) form by attaching a label to the nucleic acid proliferation template during the RPA reaction or labelling at the detector to enhance the signal [23], allowing visual confirmation of the results [24].

Therefore, this study developed rapid PEDV detection using the RT-RPA-NALF technique. The M gene of PEDV was targeted for RT-RPA-NALF because it encodes to the M protein, which is an envelope glycoprotein component required for viral assembly processing and induces host-antibody response [25]. In addition, the M gene of PEDV differs significantly from other coronaviruses but is highly conserved in various PEDV strains [26]. In addition, one region in the M gene cannot enable recombination between PEDV strains, but it can occur in the N gene. Thus, the M gene is a promising target for establishing various detection methods, such as ELISA, RT-qPCR, and RT-PCR [27]. Specific primer pairs and probes were designed to detect the M gene, a conserved gene of PEDV by RT-RPA. Moreover, the lateral flow assay was combined with this rapid PEDV detection test to increase sensitivity and field applicability.

## 2. Materials and Methods

### 2.1. Viruses, Clinical Samples and Nucleic Acid Preparation

The M gene PEDV plasmid (M-pTrigx6)-infected *E. coli* DH5α and PEDV were obtained from Professor (Dr.) Wanpen Chaicumpa, Department of Parasitology, Siriraj Hospital, Mahidol University, Bangkok, Thailand. Pseudorabies (ADV, batch no. 50F9, Auskipra-gn, Laboratorios Hipra, S.A., Girona, Spain), porcine circovirus 2 (PCV2, batch no.A039A01, Intervet International B.V., Boxmeer, The Netherlands), porcine parvovirus (PPV, PARVOSENG, Laboratorios Hipra, S.A., Spain), porcine reproductive and respiratory syndrome virus (PRRS, batch no.A606BE01, Prime Pac, Intervet International B.V., Boxmeer, The Netherlands), and classical swine fever virus (CSFV batch no. 321 EHC 10, Choong Ang Vaccine Laboratories Co., Ltd. (CAVAC), Seoul, South Korea) were commercially available vaccine derived from Assoc. Prof. Kampon Kaeoket, Faculty of Veterinary Science, Mahidol University (Nakhon Pathom, Thailand).

The viruses were propagated in cell culture. Briefly, 500 μL PEDV were infected in a confluent monolayer of Vero cells (ATV CCL-81) in a flask containing a medium supplemented with 0.3% tryptone phosphate broth, 0.02% yeast extract, 1% penicillin/streptomycin and 10 μg/mL trypsin. After 24 h incubation, it was freeze-thawed three times and the media clarified by centrifugation at 10,000× *g* for 10 min. Then, the supernatant was collected and centrifuged at 20,000× *g* before being resuspended in 100 mL phosphate-buffered saline (PBS) for titer determination by immune-peroxidase monolayer assay according to Kerber’s method.

The M-pTrigx6-infected *E. coli* D5α was cultured in LB broth at 37 °C overnight with shaking at 150 rpm, and the plasmid was extracted using a Presto^TM^ mini plasmid kit (Geneaid, Taipei City, Taiwan) following the manufacturer’s instructions. DNA concentrations were measured using a Thermo Scientific Nanodrop 2000 spectrophotometer (Thermofisher Scientific, Waltham, MA, USA). The number of DNA copies was determined using the following formula: DNA copy number = (M × 6.02 × 10^23^)/((*n* × 660) × 10^9^), where M is the amount of amplicon, *n* is the length of dsDNA amplicon and 660 is the average mass of one base pair DNA molecule. The extracted M gene plasmid was used as a template for screening appropriate primer pairs using the PCR method and to optimize the annealing temperature of the RPA assay.

The porcine clinical fecal samples were obtained from Assoc. Prof. Kampon Kaeoket. The samples were diluted at 1:100 with PBS, then vortexed and centrifuged at 14,000× *g* at 4 °C for 10 min. Next, the supernatant was collected for RNA extraction with FavorPrep™ Viral DNA/RNA Kit (Favorgen, Ping-Tung, Taiwan). The extracted RNA was stored at −80 °C until use.

All research procedures for the animal part of this study were approved by the Faculty of Veterinary Sciences-Animal Care and Use Committee, Mahidol University, Thailand (Protocol no. MUSV-2021-03-09).

### 2.2. Primers and Probe Design

Based on the conserved M gene of PEDV (Accession no. JX435310), the RT-qPCR primer of this study was designed and verified by BLAST analysis (https://blast.ncbi.nlm.nih.gov/Blast.cgi; accessed on 1 June 2021) and synthesized by SBS Genetech, Beijing, China. For the RT-RPA-NALF assay, four forward and four reverse primers were designed following the TwistDx design manual for RPA (TwistDx Inc., Cambridge, UK) and synthesized by SBS Genetech. The probe was designed following the manual guide instrument for use in lateral flow based on the sequencing primer and was conjugated with the fluorescein amidites (FAM) and the black hole quencher to the T-bases at internal positions with a THF located in the central part of the two fluorescent groups and an SpC3 labelled on the 3′ end. The specificity of each primer pair was investigated using Primer-BLAST software (https://www.ncbi.nlm.nih.gov/tools/primer-blast/; accessed on 1 June 2021). A suitable reverse primer for lateral flow was synthesized and biotin-labelled. The primer and probe sequences are shown in Table 1.

### 2.3. RT-qPCR

RT-qPCR reaction was performed using KAPA SYBR^®^ FAST One-Step qRT-PCR kits (TransGene, Beijing, China). Briefly, 10 μL 2× SYBR one-step RT-qPCR reaction buffer, 0.4 μL 10 mM each forward and reverse primers, 0.4 μL Rox Low, RT mix, 5 μL RNA template and adjusted to 20 μL with molecular water. The reaction was performed following a condition from 42 °C for 30 min, 95 °C for 10 min, then 35 cycles at 95 °C for 1 min, 55 °C for 1 min, 72 °C for 1 min using an Agilent Mx3005p thermocycler machine. The temperature range for the melting curve analysis was 65 °C–95 °C, rising by 0.5 °C in each step. The established RT-qPCR also determined the sensitivity using PEDV RNA extracted from pure culture, the viral titer from 10^5^ to 10^0^ TCID_50_/mL and specificity with heterologous swine pathogens; *E. coli*, ETEC, *Salmonella* Enterica 13076, *Salmonella* Choleraesuis 10708, Enterovirus 71 B5 (EV71 B5), Enterovirus 71 C4 (EV71 C4), Enterovirus 71 BrCr (EV71 BrCr), Coxsackievirus A-16 (CVA16), ADV, PCV2, PPV, PRRS, and CSFV. The intra-and inter-test reproducibility assay of RT-qPCR was performed in triplicates by testing the samples within the same run and three times in independent runs on different days.

### 2.4. RPA Primer Screening

During the primer pair screening, 16 primer pairs were tested from four forward and four reverse primers were tested for their ability to amplify the M gene of PEDV by conventional PCR with the M gene plasmid as a template. The PCR reaction mixture (25 μL) contained a 3μL DNA template, 2.5 μL of 10× *Taq* buffer, 2 mM MgCl_2_, 0.2 mM dNTP, 1 μM of each primer and 1 U of *Taq* polymerase (Thermo Fisher Scientific, Waltham, MA, USA). The thermal cycles were initial denaturation at 95 °C for 5 min, 40 cycles of denaturation at 95 °C for 30 sec, annealing at 46 °C for 45 sec, extension at 72 °C for 45 sec and final extension at 72 °C for 7 min. The amplicons were electrophoresed on 1.5% (*w/v*) agarose gel in 1× TAE buffer (tris-acetate-EDTA) and stained with ethidium bromide. DNA bands were visualized using the ChemiDoc MP imaging system (Bio-Rad, Hercules, CA, USA). Four primer pairs that yielded high amplicon and specificity were selected for RPA reaction optimization using the M gene plasmid as template. The RPA reaction was performed with a TwistAmp^TM^ liquid exo kit (TwistDx Inc., Cambridge, UK) following the manufacturer’s instructions. The mixture reaction (50 μL) contained 0.6 μL of 10 μM probe, 2.1 μL 10 μM of each primer, 5 μL 10× Probe E-mix, 25 μL of 2× Reaction buffer, 2.5 μL of 20× core reaction mix, 1 μL 50× Exo and 1 μL of a template. The reaction was initiated by adding 2.5 μL of magnesium acetate. The RPA reaction was performed at 40 °C for 20 min and immediately measured with the FAM channel (excitation, 470 nm; detection, 520 nm) in an Agilent Mx3005p thermocycler. The primer pair that yields high fluorescence signal (dR) by RPA and specificity by PCR was selected and optimized at an annealing temperature of 40 °C, 41 °C and 42 °C for 20 min. The selected primer pair and the optimized temperature were used for PEDV detection by RT-RPA.

### 2.5. RT-RPA and RT-RPA-NALF

RT-RPA was performed using PEDV RNA as template and the selected primer pair. The reaction mixture was prepared using the TwistAmp^TM^ liquid exo kit (TwistDx) as mentioned above, with the addition of 10 U Transcriptor (Thermo Fisher Scientific, Waltham, MA, USA), 20 U Ribolock RNase inhibitor (Thermo Fisher Scientific, Waltham, MA, USA), 2 mM DTT and 1 μL viral RNA or extracted RNA sample. The RT-RPA reaction tubes were immediately placed in an Agilent Mx3005p thermocycler to start the reaction at 42 °C for 20 min (20 s per cycle for 60 cycles).

For NALF detection of the RT-RPA products, the selected reverse primer for RT-RPA was labelled at the 5′-end with biotin and the probe tags with FAM. After amplification, RT-RPA products were diluted at 1:10 with the running buffer and the lateral flow strip, Milenia Genline HybriDetect LFD (Milenia Biotec, Giessen, Germany), was vertically inserted into 200 μL of the diluted product at room temperature. The result was read within 10 min and was considered valid if a black line at the control was visible, and positive when a black line appeared at both the Control and Test lines.

### 2.6. Evaluation of the RT-RPA-NALF

Based on the optimized conditions for RT-RPA-NALF, the sensitivity of this assay was evaluated and compared with RT-qPCR using PEDV RNA extracted from pure culture and the viral titer from 10^5^ to 10^1^ TCID_50_/mL. To validate cross-reactivity, different swine pathogens were subjected to this experiment. To simulate the field-detecting condition, five porcine fecal samples considered negative for PEDV by RT-qPCR, were diluted at 1:10 in 1× PBS and spiked with 10^3^ TCID_50_ of PEDV, then vortexed and centrifuged at 14,000× *g* at 4 °C for 10 min. The supernatants were collected for nucleic acid extraction using FavorPrep^TM^ Viral DNA/RNA kit, following the manufacturer’s instructions. Extracted RNA was subjected to performance evaluation in RT-RPA-NALF compared with RT-qPCR. Sixty clinical fecal samples from swine farms were collected and used to evaluate the diagnostic validity of RT-RPA-NALF. Agreement between RT-RPA-NALF assay and RT-qPCR assay was assessed with receiver operating characteristic (ROC) analysis by using SPSS software version 23.0. The level of agreement was calculated as the area under the curve (AUC). Moreover, to evaluate the reproducibility of the RPA-NALF method, intra-assay and inter-assay reproducibility were determined by running the same standard samples with three replicates independent times.

## 3. Results

### 3.1. Establishment of the PEDV RT-qPCR

**Figure 1:**
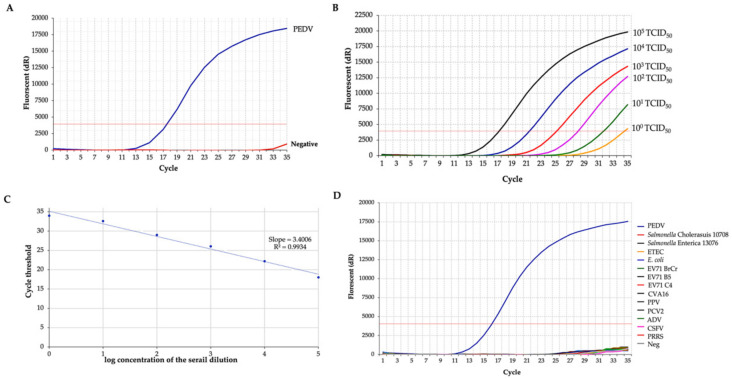
RT-qPCR for PEDV. (**A**) M gene amplification with PEDV. (**B**) PEDV RT-qPCR with other porcine enteric pathogens. (**C**) Sensitivity of PEDV RT-qPCR assay. (**D**) Specificity of PEDV RT-qPCR assay. Negative: the reaction mixture with no template.

In this study, a RT-qPCR was established. The developed RT-qPCR showed a positive fluorescence signal of M gene amplification for the extracted PEDV RNA (Figure 1A) and melting-curve analysis is shown in Supplementary Data S2. To assess sensitivity, PEDV RNA was extracted from viral titers ranging from 10^5^ TCID_50_/mL to 10^0^ TCID_50_/mL and analyzed by the developed RT–qPCR technique. The results demonstrated that the limitation for PEDV determination was 10^1^ TCID_50_/mL (Figure 1B). A standard curve (Figure 1C) was obtained in which the coefficient of determination (*R*^2^) was >0.99, and the slope of the curve was 3.4006, indicating the high PCR efficiency of the experiment. The specificity of the established RT-qPCR was determined by analyzing eight different heterologous swine enteric pathogens. Positive results were found in the PEDV sample, but no positive fluorogenic signal was observed in the interpreted cycle for heterologous agents (Figure 1D). A Ct value less than 30 is interpreted as a positive result.

### 3.2. Establishment of the PEDV RT-RPA-NALF

**Figure 2:**
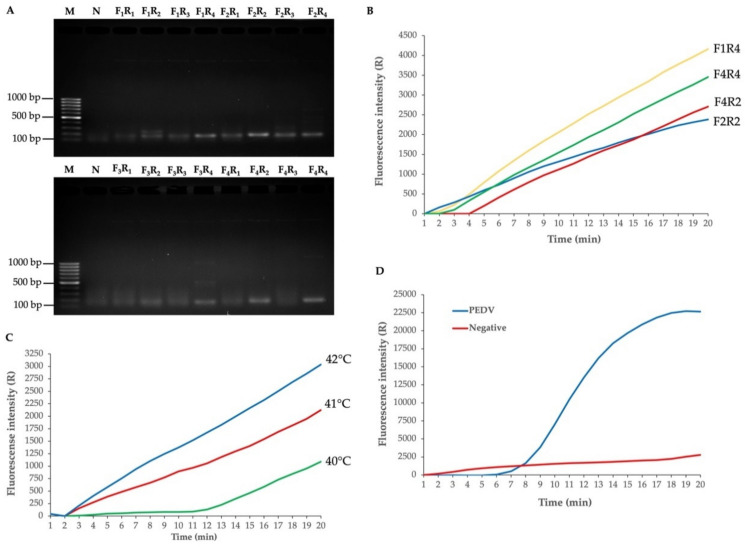
Optimization for M gene of PEDV amplification. (**A**) Screening primer pair for M gene amplification by conventional PCR. M: DNA marker; N: negative, no template; Primer pair: F1R1, F1R2, F1R3, F1R4, F2R1, F2R2, F2R3, F2R4, F3R1, F3R2, F3R3, F3R4, F4R1, F4R2, F4R3, F4R4. (**B**) Amplification of M gene by RPA. (**C**) Optimization of RPA condition. (**D**) RT-RPA M gene amplification with PEDV RNA template. Negative: the reaction mixture with no template.

The primer pair is essential in the M gene for PEDV amplification. Therefore, the primer pair was screened for usability to amplify the M gene of PEDV with 16 primer sets by conventional PCR. The results showed that the 170-bp target M gene could be successfully amplified (Figure 2A). The four selected primer pairs (F1:R4; F2:R2; F4:R2; F4:R4), which showed high intensity PCR amplicon following agarose gel analysis, were used to amplify the M gene from the plasmid by the RPA assay. The results showed that all four selected primer pairs yielded high amplification fluorescence signals by RPA (Figure 2B). However, the PCR amplicon using the F2:R2 primer pair showed high band intensity, which indicated a high-yield amplicon among the four primer combinations (Figure 1A,B). This enhances the sensitivity of the NALF. Thus, the F2:R2 primer pair was chosen, and the annealing temperature was optimized. The results showed that a temperature of 42 °C showed a high amplification fluorescence signal by RPA (Figure 2C). Therefore, the F2:R2 primer pair and annealing temperature at 42 °C for 20 min was used to amplify the M gene by RT-RPA assay. By RT-RPA, M gene amplification from the extracted PEDV RNA with the optimized conditions was achieved, yielding a significantly high amplification fluorescence signal compared with the negative control (Figure 2D). Thus, the reversed primer (R2) was selected for labelling with biotin at the 5′end for use in NALF detection. The relative conservation of F2:R2 primer and probe for M regions relative to other regions of the PEDV genome was defined and the results showed that all sequences have 100% percent identity to M gene region and less than 10 bp match to another region of PEDV genome (Supplementary Data S3–S5).

**Figure 3:**
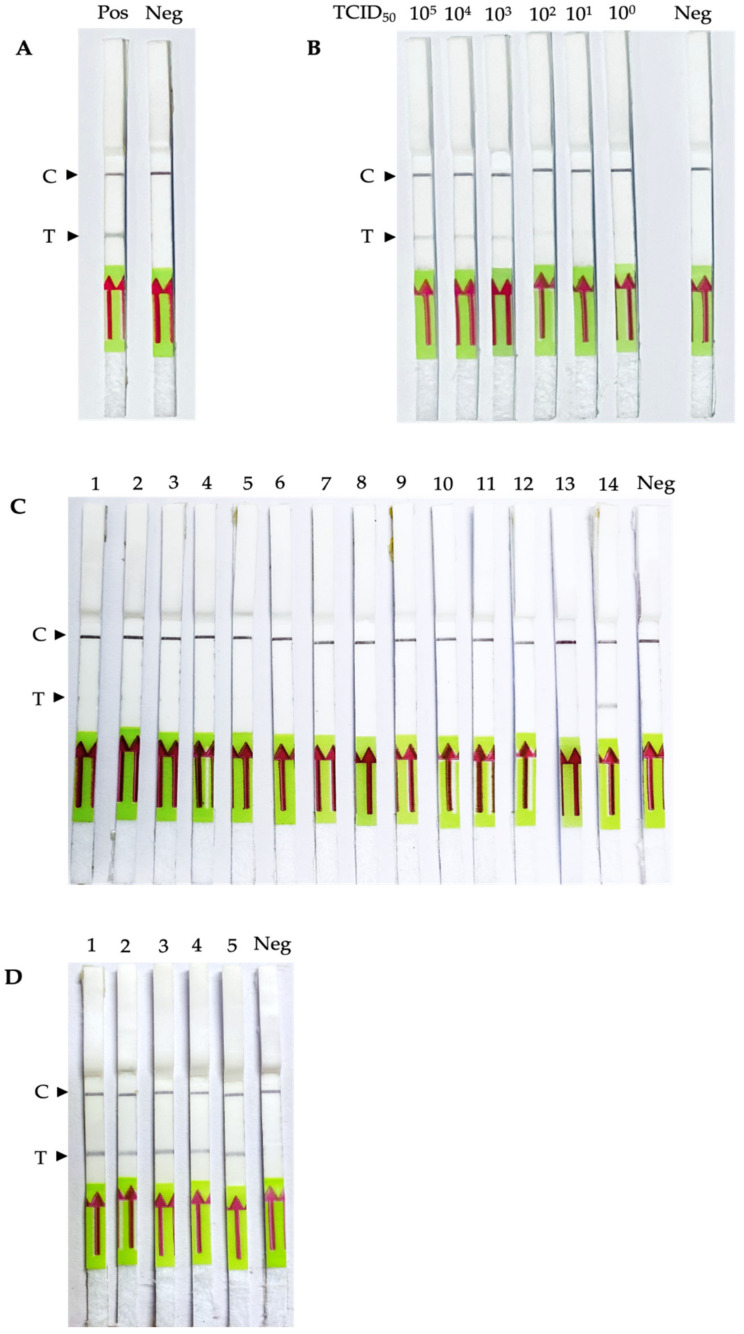
Sensitivity, Specificity and Usability of PEDV RT-RPA-NALF assay. (**A**) PEDV detection by RT-RPA-NALF. Pos: RT-RPA product from extracted PEDV RNA; Neg: negative control, RT-RPA product without template. (**B**) Sensitivity of the RT-RPA-NALF assay. To determine the detection limit of the RT-RPA-NALF assay, tenfold serial dilutions of the RNA standard ranging from 10^5^ TCID_50_/mL to 10^0^ TCID_50_/mL (varied from 10^5^, 10^4^, 10^3^, 10^2^, 10^1^, and 10^0^; Neg: negative control) were evaluated. (**C**) Specificity test of the RT-RPA-NALF assay. The nucleic acids of 14 common swine pathogens were used to validate the cross-reactivity of the RT-RPA-LFD assay and replicated testing for each. Strip no.1–14: *E. coli*, ETEC, *Salmonella* Enterica 13076, *Salmonella* Choleraesuis 10708, Enterovirus 71 B5 (EV71 B5), Enterovirus 71 C4 (EV71 C4), Enterovirus 71 BrCr (EV71 BrCr), Coxsackievirus A16 (CVA16), Pseudorabies (ADV), porcine circovirus 2 (PCV2), porcine parvovirus (PPV), porcine reproductive and respiratory syndrome virus (PRRS), classical swine fever virus (CSFV), porcine epidemic diarrhea virus (PEDV), respectively; Neg: the reaction mixture with no template (**D**) The usability of PEDV RT-RPA-NALF in simulated PEDV clinical fecal samples. Strip no.1–5: simulated PEDV clinical fecal samples no.1–5; Neg: the reaction mixture with no template, C: Control line; T: Test line.

The usability of PEDV RT-RPA-NALF was investigated with extracted PEDV RNA. The RPA condition was set as an incubation temperature of 42 °C and an incubation time of 20 min. Diluted RT-RPA products were applied to the NALF strip, and the Test and Control lines were observed. The results showed that PEDV RT-RPA-NALF was positive for the PEDV sample and negative for the negative control (Figure 3A).

### 3.3. Sensitivity, Specificity and Usability of the PEDV RT-RPA-NALF

The sensitivity of PEDV RT-RPA-NALF was determined with PEDV RNA extracted from the viral titer 10^5^–10^0^ TCID_50_/mL. In Figure 3B, the detection limit was 10^2^ TCID_50_/mL. To evaluate the specificity of PEDV RT-RPA-NALF, heterologous swine pathogens were used as templates for this assay. In Figure 3C, except for the PEDV sample, only a black control line was observed on the NALF strip. The results showed that PEDV RT-RPA-NALF could discriminate PEDV from other porcine pathogens. Five simulated PEDV fecal samples were used to determine the usability of PEDV RT-RPA-NALF in a clinical fecal sample. The results showed that all five simulated samples were positive (Figure 3D). These results indicate that the established PEDV RT-RPA-NALF can efficiently determine PEDV in fecal specimens. The repeatability of the RPA-NALF assay was investigated using different standard control sample concentrations (10^5^, 10^4^, 10^3^, 10^2^, 10^1^, and 10^0^ TCID_50_/mL) under the same conditions, and the assay demonstrated correlated results for inter-and intra-assay repeated experiments (Supplementary Data S1).

### 3.4. Validation of Simulated and Clinical Samples

**Figure 4:**
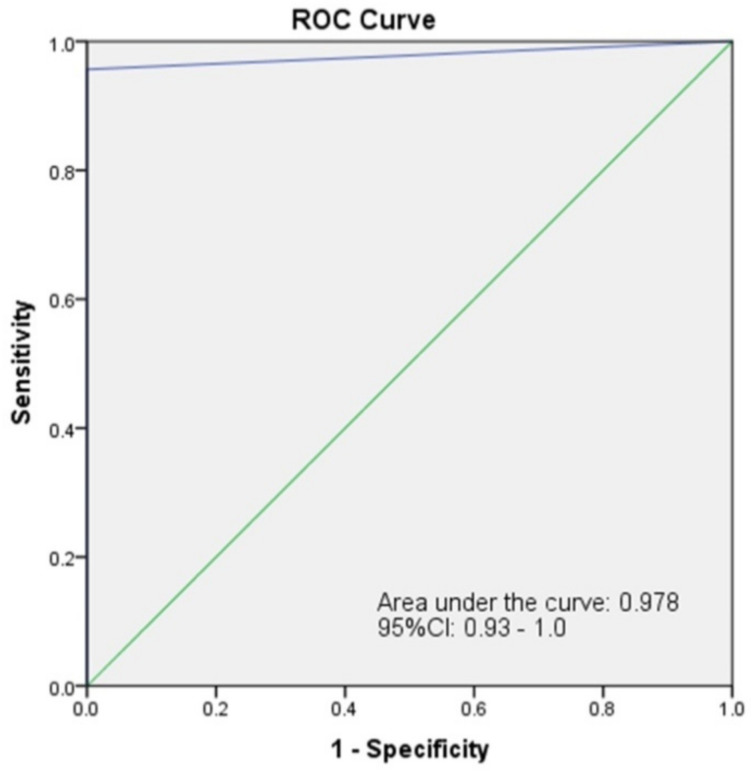
ROC analysis of the agreement between the RT-RPA-NALF assay and RT-qPCR assay.

Validation of the PEDV RT-RPA-NALF assay compared with RT-qPCR was evaluated using 60 clinical swine fecal samples collected from a swine farm. The results showed that 23 samples were PEDV-positive, whereas others were negative by RT-qPCR. Among the 23 positive samples, 22 were PEDV-positive by PEDV RT-RPA-NALF, while 38 samples were PEDV-negative by this assay. The sensitivity and specificity of RT-RPA-NALF were 95.65% and 100%, respectively (Table 2). The coincident rate of the RT-RPA-NALF and RT-qPCR assays was 98.33%, indicating that the PEDV RT-PEDV is effective for detecting PEDV. The ROC analysis of the agreement of RT-RPA-NALF assay with RT-qPCR assay produce an AUC of 0.978, with a 95% confidence interval between 0.929–1.0, signifying excellent agreement between the two methods (Figure 4).

## 4. Discussion

PEDV is a significant contagious organism that can cause gastroenteritis in suckling and weaned piglets, and can spread rapidly worldwide [28,29,30]. Presently, a disease acquired from animals, particularly a virus-causing infectious disease, is one of the factors affecting the consistency of swine production, contributing to heavy global economic losses at the industrial level. Although several strategies have been established to prevent and control these infectious diseases, schemes for cost-effective and rapid diagnosis are still being developed. Simple, rapid diagnosis with high sensitivity and specificity is crucial for the surveillance, control and prevention of the spread of animal diseases, especially field diagnosis, which is necessary to diminish virus expansion, financial losses and disease transmission to humans. Ideally, for early PEDV detection, PCR-based assays can identify a viral genome in infected animals at the onset of the disease (pre-clinical), but it still requires sophisticated laboratory apparatus and well-trained workers. Various methods have been considered as “diagnostics-in-a-suitcase” for investigating several human and animal pathogens using isothermal amplification and RPA-based technology applied for nucleic acid detection [15,16,17,18,19,20,21,22]. However, the benefit of the RT-RPA-based technique over traditional RT-qPCR is the sufferance for PCR amplification inhibitors, which has been demonstrated by earlier studies [31,32]. Recent study established RT-RPA-LFD assay to detect PEDV within the conserve N gene with high specificity [33]. It indicates that the conserve region is an appropriated target for developing the diagnostic detection of PEDV.

In this study, only one biotin-labelled primer and one FAM-labelled probe were used. The RT-RPA reaction produced a duplex-tagged DNA amplicon that could be envisaged on a portable NALF strip. The RT-RPA-NALF assay developed in this study has some sensitivity and specificity. The assay could detect even 10^2^ TCID_50_/mL of PEDV genomic copies and exhibited no cross-reaction with other porcine infectious agents. The assay was accomplished at a one-point temperature (42 °C) and used a short reaction time of approximately 25 min (less than 30 min) in a metal or water bath. The target of the primers for the RT-RPA-NALF assay was the M gene-conserved sequence. Herein, in the 23 PEDV-positive clinical samples determined using RT-qPCR, 22 were positive for the clinical PEDV specimens by the RT-RPA-NALF assay. This new assay postulates higher sensitivity (95.65%) and specificity (100%) than those in previous research [33,34,35], which indicates the reliability and accuracy of this diagnostic test kit. Though the limitation of this RT-RPA-NALF assay was its inability to distinguish wild-type strains from the vaccine strain, the test results in this study and the aforementioned reports both indicate that the wild-type virus was commonly found to be the mainstream of clinical specimens, whereas the number of clinical samples containing the vaccine strains was quite low. Essentially, differentiation of the wild-type strains from a vaccine strain is less critical than the rapid detection of the infectious pathogen. The urgent detection of PEDV upon the indication of clinical signs and symptoms of pig diarrhoea in a swine farm, particularly in rural limited-equipped areas, is essential for the immediate handling and operation of treatment measures.

Therefore, the established RT-RPA technique combined with NALF is a proficient, possibly point-of-care (POC) diagnostic that is more cost-effective and easier to operate in field settings than the conventional RT-qPCR assay, thus offering a considerable new paradigm for PEDV monitoring and control in resource-limited locations.

## 5. Conclusions

This study shows conclusively that the developed RT-RPA-NALF assay, targeting the highly conserved M gene for PEDV, is simple, quick and cost-effective for the on-site screening of clinical fecal samples. Thus, this can serve as a POC diagnostic tool for the accurate and rapid detection of this animal pathogenic virus under field settings to enterprise active surveillance and instant effective control measures.

## Figures and Tables

**Table 1:** Primer and probe sequences for porcine RT-qPCR and RT-RPA-NALF assay used in this study.

Assay	Primers and Probe	Sequence 5′–3′	Product Size (bp)
RT-qPCR	MPEDVF	ATGTCTAACGGTTCTATTCCC	113
	MPEDVR	TAATGGCCATACTGAAGCAC	
RT-RPA-NALF	F1	CTGTGATGGGCCGACAGGTCTGCATTCCAG	170
	F2	CTGTGATGGGCCGACAGGTCTGCATTCCAGTG	
	F3	CTGTGATGGGCCGACAGGTCTGCATTCCAGTGC	
	F4	CTGTGATGGGCCGACAGGTCTGCATTCCAGTGCTTG	
	R1	GACAATTGTTGTAGTGGCCTTGGCGACTG	
	R2	GACAATTGTTGTAGTGGCCTTGGCGACTGTG	
	R3	ACAATTGTTGTAGTGGCCTTGGCGACTGTGAC	
	R4	GACAATTGTTGTAGTGGCCTTGGCGACTGTGACG	
	Probe	CTGGTGTAACGCTAACACTCCTTAGTGG [FAM-dT] A [THF] A [BHQ-1-DT] TGCTTGTAGAGCG [3PHOS]	

**Table 2:** Comparison of RT-RPA-NALF and RT-qPCR.

	RT-qPCR		Total	^1^ Coincident Rate	^2^ Sensitivity	^3^ Specificity
	Positive	Negative				
RT-RPA-NALF				98.33%	95.65%	100%
RT-RPA-NALF Positive	22	0	22			
Negative	1	37	38			
Total	23	37	60			

^1^ Coincidence rate = ((22 + 37)/60) × 100%, ^2^ Sensitivity = (22/23) × 100%, ^3^ Specificity = (37/37) × 100%

## Supplementary Materials

The following supporting information can be downloaded at: https://www.mdpi.com/article/10.3390/biology11071018/s1, Supplementary Data S1: Reproducibility of the RPA-NALF assay.; Supplementary Data S2: Melting curve analysis of M gene amplification by RT-qPCR.; Supplementary Data S3: The relative conservation of F2 primer for M regions relative to other regions of the PEDV genome.; Supplementary Data S4: The relative conservation of R2 primer for M regions relative to other regions of the PEDV genome.; Supplementary Data S5: The relative conservation of probe for M regions relative to other regions of the PEDV genome.
