# Supplementary material for: Development of a Rapid Reverse Transcription-Recombinase Polymerase Amplification Couple Nucleic Acid Lateral Flow Method for Detecting Porcine Epidemic Diarrhoea Virus
Source: Biology (Basel). 2022 Jul 6;11(7):1018. doi: 10.3390/biology11071018 (PMC9312133; doi:10.3390/biology11071018)
Supplement: Supplementary file 1 [file biology-11-01018-s001.zip › Supplementary Data S1.pdf]

Supplementary Data S1. Reproducibility of the RPA-NALF assay.

Concentration of RNA standard (TCID$_{50}$/mL)	Positive number (Intra-assay, n = 3)	Positive number (Inter-assay, n = 3)
10^5	3	3
10^4	3	3
10^3	3	3
10^2	3	2
10^1	2	1
10^0	1	1
