# Supplementary material for: Development of a Rapid Reverse Transcription-Recombinase Polymerase Amplification Couple Nucleic Acid Lateral Flow Method for Detecting Porcine Epidemic Diarrhoea Virus
Source: Biology (Basel). 2022 Jul 6;11(7):1018. doi: 10.3390/biology11071018 (PMC9312133; doi:10.3390/biology11071018)
Supplement: Supplementary file 1 [file biology-11-01018-s001.zip › Supplementary Data S3.pdf]

BLAST ® » blastn suite » results for RID-BR857EF1016

Your search is limited to records that include: Porcine epidemic diarrhea virus (taxid:28295)

Job Title	F2 ...
RID	BR857EF1016 Search expires on 06-30 08:45 am
Program	BLASTN
Database	ref_viruses_rep_genomes
Query ID	lcl|Query_65179
Description	F2 ...
Molecule type	dna
Query Length	32

Descriptions

Descriptions

Description	Scientific Name	Max Score	Total Score	Query Cover	E value	Per. Ident	Acc. Len	Accession
Porcine epidemic diarrhea virus, complete genome	Porcine epidemic diarrhea virus	63.9	1037	100%	4e-14	100.00%	28033	NC_003436.1

Graphic Summary

Distribution of the top 65 Blast Hits on 1 subject sequences

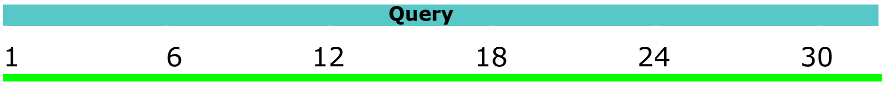

Alignments

Alignment view Pairwise CDS feature Restore defaults

Porcine epidemic diarrhea virus, complete genome

Sequence ID: **NC_003436.1** Length: 28033 Number of Matches: 65

Range 1: 26049 to 26080

Score	Expect	Identities	Gaps	Strand	Frame
63.9 bits(32)	4e-14()	32/32(100%)	0/32(0%)	Plus/Plus	

```
Query  1      CTGTGATGGGCCGACAGGTCTGCATTCC
              ||||||||||||||||||||||||||||||||
Sbjct  26049  CTGTGATGGGCCGACAGGTCTGCATTC
```

Range 2: 7086 to 7095

Score	Expect	Identities	Gaps	Strand	Frame
20.3 bits(10)	0.49()	10/10(100%)	0/10(0%)	Plus/Plus	

```
Query  11    CCGAC
             ||||||||||
Sbjct  7086
```

Range 3: 7430 to 7438

Score	Expect	Identities	Gaps	Strand	Frame
18.3 bits(9)	2.0()	9/9(100%)	0/9(0%)	Plus/Minus	

```
Query  13    GACA
             |||||||||
Sbjct  7438
```

Range 4: 18418 to 18426

Score	Expect	Identities	Gaps	Strand	Frame
18.3 bits(9)	2.0()	9/9(100%)	0/9(0%)	Plus/Plus	

```
Query  21     TGCA
              |||||||||
Sbjct  1841
```

Range 5: 19917 to 19925

Score	Expect	Identities	Gaps	Strand	Frame
18.3 bits(9)	2.0()	9/9(100%)	0/9(0%)	Plus/Minus	

```
Query  2      TGTGA
              |||||||||
Sbjct  1992
```

Range 6: 20147 to 20155

Score	Expect	Identities	Gaps	Strand	Frame
18.3 bits(9)	2.0()	9/9(100%)	0/9(0%)	Plus/Plus	

```
Query  2     TGTGA
             |||||||||
Sbjct  2014
```

Range 7: 26528 to 26536

Score	Expect	Identities	Gaps	Strand	Frame
18.3 bits(9)	2.0()	9/9(100%)	0/9(0%)	Plus/Minus	

```
Query  23    CATT
             |||||||||
Sbjct  2653
```

Range 8: 2341 to 2348

Score	Expect	Identities	Gaps	Strand	Frame
16.4 bits(8)	7.7()	8/8(100%)	0/8(0%)	Plus/Minus	

```
Query  6     ATGG
             ||||||||
Sbjct  234
```

Range 9: 5889 to 5896

Score	Expect	Identities	Gaps	Strand	Frame
16.4 bits(8)	7.7()	8/8(100%)	0/8(0%)	Plus/Minus	

```
Query  2     TGT
             ||||||||
Sbjct  589
```

Range 10: 5951 to 5958

Score	Expect	Identities	Gaps	Strand	Frame
16.4 bits(8)	7.7()	8/8(100%)	0/8(0%)	Plus/Minus	

```
Query  9     GGCC
             ||||||||
Sbjct  595
```

Range 11: 6455 to 6462

Score	Expect	Identities	Gaps	Strand	Frame
16.4 bits(8)	7.7()	8/8(100%)	0/8(0%)	Plus/Plus	

```
Query  1     CTG
             ||||||||
Sbjct  645
```

Range 12: 8544 to 8551

Score	Expect	Identities	Gaps	Strand	Frame
16.4 bits(8)	7.7()	8/8(100%)	0/8(0%)	Plus/Minus	

```
Query  19    TCT
             ||||||||
Sbjct  855
```

Range 13: 13153 to 13160

Score	Expect	Identities	Gaps	Strand	Frame
16.4 bits(8)	7.7()	8/8(100%)	0/8(0%)	Plus/Plus	

```
Query  1     CTG
```

```
Sbjct  131     ||||||||
```

Range 14: 15613 to 15620

Score	Expect	Identities	Gaps	Strand	Frame
16.4 bits(8)	7.7()	8/8(100%)	0/8(0%)	Plus/Plus	

```
Query  21      TGC
               ||||||||
Sbjct  156
```

Range 15: 17070 to 17077

Score	Expect	Identities	Gaps	Strand	Frame
16.4 bits(8)	7.7()	8/8(100%)	0/8(0%)	Plus/Minus	

```
Query  2       TGT
               ||||||||
Sbjct  170
```

Range 16: 17524 to 17531

Score	Expect	Identities	Gaps	Strand	Frame
16.4 bits(8)	7.7()	8/8(100%)	0/8(0%)	Plus/Minus	

```
Query  15      CAG
               ||||||||
Sbjct  175
```

Range 17: 18042 to 18049

Score	Expect	Identities	Gaps	Strand	Frame
16.4 bits(8)	7.7()	8/8(100%)	0/8(0%)	Plus/Minus	

```
Query  2       TGT
               ||||||||
Sbjct  180
```

Range 18: 21081 to 21088

Score	Expect	Identities	Gaps	Strand	Frame
16.4 bits(8)	7.7()	8/8(100%)	0/8(0%)	Plus/Plus	

```
Query  23      CAT
               ||||||||
Sbjct  210
```

Range 19: 25731 to 25738

Score	Expect	Identities	Gaps	Strand	Frame
16.4 bits(8)	7.7()	8/8(100%)	0/8(0%)	Plus/Minus	

```
Query  24      ATT
               ||||||||
Sbjct  257
```

Range 20: 27503 to 27510

Score	Expect	Identities	Gaps	Strand	Frame
16.4 bits(8)	7.7()	8/8(100%)	0/8(0%)	Plus/Minus	

```
Query  21      TGC
               ||||||||
Sbjct  275
```

Range 21: 27622 to 27629

Score	Expect	Identities	Gaps	Strand	Frame
16.4 bits(8)	7.7()	8/8(100%)	0/8(0%)	Plus/Minus	

```
Query  23    CAT
             ||||||||
Sbjct  276
```

Range 22: 334 to 340

Score	Expect	Identities	Gaps	Strand	Frame
14.4 bits(7)	30()	7/7(100%)	0/7(0%)	Plus/Minus	

```
Query  19   TC
            |||||||
Sbjct  340  TC
```

Range 23: 1817 to 1823

Score	Expect	Identities	Gaps	Strand	Frame
14.4 bits(7)	30()	7/7(100%)	0/7(0%)	Plus/Minus	

```
Query  7     TGG
             |||||||
Sbjct  18
```

Range 24: 1824 to 1830

Score	Expect	Identities	Gaps	Strand	Frame
14.4 bits(7)	30()	7/7(100%)	0/7(0%)	Plus/Plus	

```
Query  12    CG
             |||||||
Sbjct  18
```

Range 25: 1985 to 1991

Score	Expect	Identities	Gaps	Strand	Frame
14.4 bits(7)	30()	7/7(100%)	0/7(0%)	Plus/Plus	

```
Query  21    TG
             |||||||
Sbjct  19
```

Range 26: 3715 to 3721

Score	Expect	Identities	Gaps	Strand	Frame
14.4 bits(7)	30()	7/7(100%)	0/7(0%)	Plus/Minus	

```
Query  10    GC
             |||||||
Sbjct  37
```

Range 27: 5539 to 5545

Score	Expect	Identities	Gaps	Strand	Frame
14.4 bits(7)	30()	7/7(100%)	0/7(0%)	Plus/Plus	

```
Query  15    CA
             |||||||
Sbjct  55
```

Range 28: 6529 to 6535

Score	Expect	Identities	Gaps	Strand	Frame
14.4 bits(7)	30()	7/7(100%)	0/7(0%)	Plus/Plus	

```
Query  16    AG
             |||||||
Sbjct  65
```

Range 29: 6617 to 6623

Score	Expect	Identities	Gaps	Strand	Frame
14.4 bits(7)	30()	7/7(100%)	0/7(0%)	Plus/Plus	

```
Query  2     TG
             |||||||
Sbjct  66
```

Range 30: 6696 to 6702

Score	Expect	Identities	Gaps	Strand	Frame
14.4 bits(7)	30()	7/7(100%)	0/7(0%)	Plus/Plus	

```
Query  25    TT
             |||||||
Sbjct  66
```

Range 31: 7236 to 7242

Score	Expect	Identities	Gaps	Strand	Frame
14.4 bits(7)	30()	7/7(100%)	0/7(0%)	Plus/Minus	

```
Query  1     CT
             |||||||
Sbjct  72
```

Range 32: 7289 to 7295

Score	Expect	Identities	Gaps	Strand	Frame
14.4 bits(7)	30()	7/7(100%)	0/7(0%)	Plus/Minus	

```
Query  21    TG
             |||||||
Sbjct  72
```

Range 33: 7846 to 7852

Score	Expect	Identities	Gaps	Strand	Frame
14.4 bits(7)	30()	7/7(100%)	0/7(0%)	Plus/Plus	

```
Query  15    CA
             |||||||
Sbjct  78
```

Range 34: 8498 to 8504

Score	Expect	Identities	Gaps	Strand	Frame
14.4 bits(7)	30()	7/7(100%)	0/7(0%)	Plus/Plus	

```
Query  18    GT
             |||||||
Sbjct  84
```

Range 35: 8755 to 8761

Score	Expect	Identities	Gaps	Strand	Frame
14.4 bits(7)	30()	7/7(100%)	0/7(0%)	Plus/Minus	

```
Query  11    CC
             |||||||
Sbjct  87
```

Range 36: 9966 to 9972

Score	Expect	Identities	Gaps	Strand	Frame

14.4 bits(7)	30()	7/7(100%)	0/7(0%)	Plus/Plus	

```
Query  13    GA
             |||||||
Sbjct  99
```

Range 37: 10088 to 10094

Score	Expect	Identities	Gaps	Strand	Frame
14.4 bits(7)	30()	7/7(100%)	0/7(0%)	Plus/Plus	

```
Query  19     TC
              |||||||
Sbjct  10
```

Range 38: 10386 to 10392

Score	Expect	Identities	Gaps	Strand	Frame
14.4 bits(7)	30()	7/7(100%)	0/7(0%)	Plus/Plus	

```
Query  15     CA
              |||||||
Sbjct  10
```

Range 39: 10950 to 10956

Score	Expect	Identities	Gaps	Strand	Frame
14.4 bits(7)	30()	7/7(100%)	0/7(0%)	Plus/Minus	

```
Query  25     TT
              |||||||
Sbjct  10
```

Range 40: 11542 to 11548

Score	Expect	Identities	Gaps	Strand	Frame
14.4 bits(7)	30()	7/7(100%)	0/7(0%)	Plus/Plus	

```
Query  21     TG
              |||||||
Sbjct  11
```

Range 41: 12459 to 12465

Score	Expect	Identities	Gaps	Strand	Frame
14.4 bits(7)	30()	7/7(100%)	0/7(0%)	Plus/Minus	

```
Query  18     GT
              |||||||
Sbjct  12
```

Range 42: 12778 to 12784

Score	Expect	Identities	Gaps	Strand	Frame
14.4 bits(7)	30()	7/7(100%)	0/7(0%)	Plus/Plus	

```
Query  21     TG
              |||||||
Sbjct  12
```

Range 43: 13391 to 13397

Score	Expect	Identities	Gaps	Strand	Frame
14.4 bits(7)	30()	7/7(100%)	0/7(0%)	Plus/Minus	

```
Query  24     AT
              |||||||
Sbjct  13
```

Range 44: 15032 to 15038

Score	Expect	Identities	Gaps	Strand	Frame
14.4 bits(7)	30()	7/7(100%)	0/7(0%)	Plus/Minus	

```
Query  19     TC
              |||||||
Sbjct  15
```

Range 45: 15507 to 15513

Score	Expect	Identities	Gaps	Strand	Frame
14.4 bits(7)	30()	7/7(100%)	0/7(0%)	Plus/Minus	

```
Query  3      GT
              |||||||
Sbjct  15
```

Range 46: 15885 to 15891

Score	Expect	Identities	Gaps	Strand	Frame
14.4 bits(7)	30()	7/7(100%)	0/7(0%)	Plus/Minus	

```
Query  17     GG
              |||||||
Sbjct  15
```

Range 47: 18280 to 18286

Score	Expect	Identities	Gaps	Strand	Frame
14.4 bits(7)	30()	7/7(100%)	0/7(0%)	Plus/Plus	

```
Query  4      TGA
              |||||||
Sbjct  18
```

Range 48: 18550 to 18556

Score	Expect	Identities	Gaps	Strand	Frame
14.4 bits(7)	30()	7/7(100%)	0/7(0%)	Plus/Plus	

```
Query  20     CT
              |||||||
Sbjct  18
```

Range 49: 20001 to 20007

Score	Expect	Identities	Gaps	Strand	Frame
14.4 bits(7)	30()	7/7(100%)	0/7(0%)	Plus/Minus	

```
Query  26     TC
              |||||||
Sbjct  20
```

Range 50: 20088 to 20094

Score	Expect	Identities	Gaps	Strand	Frame
14.4 bits(7)	30()	7/7(100%)	0/7(0%)	Plus/Minus	

```
Query  13     GA
              |||||||
Sbjct  20
```

Range 51: 20388 to 20394

Score	Expect	Identities	Gaps	Strand	Frame
14.4 bits(7)	30()	7/7(100%)	0/7(0%)	Plus/Plus	

```
Query  1
```

```
Sbjct  20      |||||||
```

Range 52: 21052 to 21058

Score	Expect	Identities	Gaps	Strand	Frame
14.4 bits(7)	30()	7/7(100%)	0/7(0%)	Plus/Plus	

```
Query  14      AC
               |||||||
Sbjct  21
```

Range 53: 21098 to 21104

Score	Expect	Identities	Gaps	Strand	Frame
14.4 bits(7)	30()	7/7(100%)	0/7(0%)	Plus/Plus	

```
Query  3       GT
               |||||||
Sbjct  21
```

Range 54: 21117 to 21123

Score	Expect	Identities	Gaps	Strand	Frame
14.4 bits(7)	30()	7/7(100%)	0/7(0%)	Plus/Minus	

```
Query  10      GC
               |||||||
Sbjct  21
```

Range 55: 22018 to 22024

Score	Expect	Identities	Gaps	Strand	Frame
14.4 bits(7)	30()	7/7(100%)	0/7(0%)	Plus/Plus	

```
Query  2       TG
               |||||||
Sbjct  22
```

Range 56: 22326 to 22332

Score	Expect	Identities	Gaps	Strand	Frame
14.4 bits(7)	30()	7/7(100%)	0/7(0%)	Plus/Minus	

```
Query  1       CT
               |||||||
Sbjct  22
```

Range 57: 22373 to 22379

Score	Expect	Identities	Gaps	Strand	Frame
14.4 bits(7)	30()	7/7(100%)	0/7(0%)	Plus/Minus	

```
Query  13      GA
               |||||||
Sbjct  22
```

Range 58: 22540 to 22546

Score	Expect	Identities	Gaps	Strand	Frame
14.4 bits(7)	30()	7/7(100%)	0/7(0%)	Plus/Minus	

```
Query  1       CT
               |||||||
Sbjct  22
```

Range 59: 23234 to 23240

Score	Expect	Identities	Gaps	Strand	Frame
14.4 bits(7)	30()	7/7(100%)	0/7(0%)	Plus/Plus	

```
Query  3     GT
             |||||||
Sbjct  23
```

Range 60: 23308 to 23314

Score	Expect	Identities	Gaps	Strand	Frame
14.4 bits(7)	30()	7/7(100%)	0/7(0%)	Plus/Plus	

```
Query  16    AG
             |||||||
Sbjct  23
```

Range 61: 24032 to 24038

Score	Expect	Identities	Gaps	Strand	Frame
14.4 bits(7)	30()	7/7(100%)	0/7(0%)	Plus/Plus	

```
Query  3     GT
             |||||||
Sbjct  24
```

Range 62: 24672 to 24678

Score	Expect	Identities	Gaps	Strand	Frame
14.4 bits(7)	30()	7/7(100%)	0/7(0%)	Plus/Plus	

```
Query  20    CT
             |||||||
Sbjct  24
```

Range 63: 25414 to 25420

Score	Expect	Identities	Gaps	Strand	Frame
14.4 bits(7)	30()	7/7(100%)	0/7(0%)	Plus/Plus	

```
Query  20    CT
             |||||||
Sbjct  25
```

Range 64: 26733 to 26739

Score	Expect	Identities	Gaps	Strand	Frame
14.4 bits(7)	30()	7/7(100%)	0/7(0%)	Plus/Plus	

```
Query  23    CA
             |||||||
Sbjct  26
```

Range 65: 28006 to 28012

Score	Expect	Identities	Gaps	Strand	Frame
14.4 bits(7)	30()	7/7(100%)	0/7(0%)	Plus/Minus	

```
Query  15    CA
             |||||||
Sbjct  28
```

Taxonomy

Reports

Lineage

Organism	Blast Name	Score	Number of Hits	Description
Porcine epidemic diarrhea virus	viruses	63.9	1	Porcine epidemic diarrhea virus hits

Organism

Description	Score	E value	Accession
Porcine epidemic diarrhea virus [viruses]			
Porcine epidemic diarrhea virus, complete genome	63.9	4e-14	NC_003436

Taxonomy

Taxonomy	Number of hits	Number of Organisms	Description
Porcine epidemic diarrhea virus	1	1	Porcine epidemic diarrhea virus hits
