# Supplementary material for: Development of a Rapid Reverse Transcription-Recombinase Polymerase Amplification Couple Nucleic Acid Lateral Flow Method for Detecting Porcine Epidemic Diarrhoea Virus
Source: Biology (Basel). 2022 Jul 6;11(7):1018. doi: 10.3390/biology11071018 (PMC9312133; doi:10.3390/biology11071018)
Supplement: Supplementary file 1 [file biology-11-01018-s001.zip › Supplementary Data S4.pdf]

Your search is limited to records that include: Porcine epidemic diarrhea virus (taxid:28295)

Job Title	R2 ...
RID	BR89R9A1016 Search expires on 06-30 08:47 am
Program	BLASTN
Database	ref_viruses_rep_genomes
Query ID	lcl|Query_4363
Description	R2 ...
Molecule type	dna
Query Length	31

Descriptions

Descriptions

Description	Scientific Name	Max Score	Total Score	Query Cover	E value	Per. Ident	Acc. Len	Accession
Porcine epidemic diarrhea virus, complete genome	Porcine epidemic diarrhea virus	58.4	58.4	100%	1e-12	100.00%	28033	NC_003436.1

Graphic Summary

Distribution of the top 1 Blast Hits on 1 subject sequences

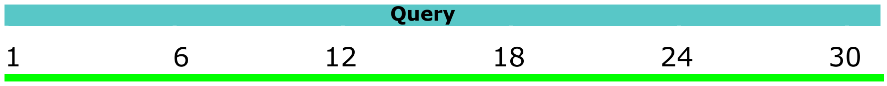

Alignments

Alignment view Pairwise CDS feature Restore defaults

Porcine epidemic diarrhea virus, complete genome

Sequence ID: **NC_003436.1** Length: 28033 Number of Matches: 1

Range 1: 26188 to 26218

Score	Expect	Identities	Gaps	Strand	Frame
58.4 bits(31)	1e-12()	31/31(100%)	0/31(0%)	Plus/Minus	

```
Query  1      GACAATTGTTGTAGTGGCCTTGGCGAC
              |||||||||||||||||||||||||||||||
Sbjct  26218  GACAATTGTTGTAGTGGCCTTGGCGA
```

Taxonomy

Reports

Lineage

Organism	Blast Name	Score	Number of Hits	Description
Porcine epidemic diarrhea virus	viruses	58.4	1	Porcine epidemic diarrhea virus hits

Organism

Description	Score	E value	Accession
Porcine epidemic diarrhea virus [viruses]			
Porcine epidemic diarrhea virus, complete genome	58.4	1e-12	NC_003436

Taxonomy

Taxonomy	Number of hits	Number of Organisms	Description
Porcine epidemic diarrhea virus	1	1	Porcine epidemic diarrhea virus hits
