# Supplementary material for: Development of a Rapid Reverse Transcription-Recombinase Polymerase Amplification Couple Nucleic Acid Lateral Flow Method for Detecting Porcine Epidemic Diarrhoea Virus
Source: Biology (Basel). 2022 Jul 6;11(7):1018. doi: 10.3390/biology11071018 (PMC9312133; doi:10.3390/biology11071018)
Supplement: Supplementary file 1 [file biology-11-01018-s001.zip › Supplementary Data S5.pdf]

The site is secure.
The **https://** ensures that you are connecting to the official website and that any information you provide is encrypted and transmitted securely.

BLAST ® » blastn suite » results for RID-BR8EG1T6013

Your search is limited to records that include: Porcine epidemic diarrhea virus (taxid:28295)

**Job Title**	Probe ...
**RID**	BR8EG1T6013 Search expires on 06-30 08:50 am
**Program**	BLASTN
**Database**	ref_viruses_rep_genomes
**Query ID**	lcl|Query_28359
**Description**	Probe ...
**Molecule type**	dna
**Query Length**	48

Descriptions

Description	Scientific Name	Max Score	Total Score	Query Cover	E value	Per. Ident	Acc. Len	Accession
Porcine epidemic diarrhea virus, complete genome	Porcine epidemic diarrhea virus	84.2	84.2	100%	4e-20	97.96%	28033	NC_003436.1

Graphic Summary

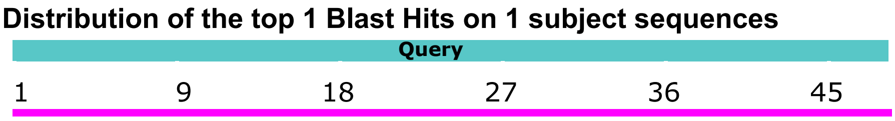

Alignments

Alignment view Pairwise

CDS feature

Restore defaults

Porcine epidemic diarrhea virus, complete genome

Sequence ID: **NC_003436.1** Length: 28033 Number of Matches: 1

Range 1: 26232 to 26280

Score	Expect	Identities	Gaps	Strand	Frame
84.2 bits(45)	4e-20()	48/49(98%)	1/49(2%)	Plus/Plus	

```
Query  1      GTCGTTCAGTCAATGCTTCATCTGGCACT-GTTGGGCTTTCTATGTCCG  48
              ||||||||||||||||||||||||||||| |||||||||||||||||||
Sbjct  26232  GTCGTTCAGTCAATGCTTCATCTGGCACTGGTTGGGCTTTCTATGTCCG  26280
```

Taxonomy

Reports

Lineage

Organism	Blast Name	Score	Number of Hits	Description
Porcine epidemic diarrhea virus	viruses	84.2	1	Porcine epidemic diarrhea virus hits

Organism

Description	Score	E value	Accession
Porcine epidemic diarrhea virus [viruses]			
Porcine epidemic diarrhea virus, complete genome	84.2	4e-20	NC_003436

Taxonomy

Taxonomy	Number of hits	Number of Organisms	Description
Porcine epidemic diarrhea virus	1	1	Porcine epidemic diarrhea virus hits
