# Supplementary figures and images for: Development of a Rapid Reverse Transcription-Recombinase Polymerase Amplification Couple Nucleic Acid Lateral Flow Method for Detecting Porcine Epidemic Diarrhoea Virus
Source: Biology (Basel). 2022 Jul 6;11(7):1018. doi: 10.3390/biology11071018 (PMC9312133; doi:10.3390/biology11071018)

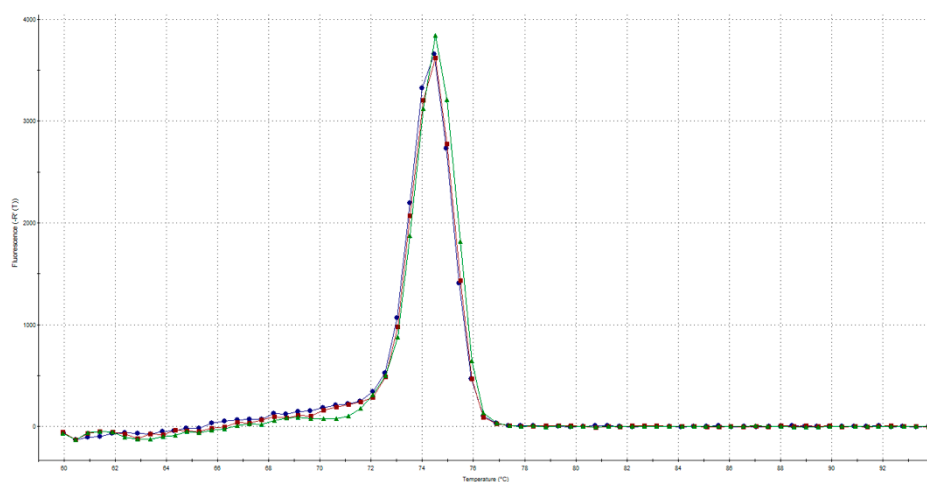

**Supplementary Data S2.** Melting curve analysis of M gene amplification by RT-qPCR.

Supplement: Supplementary file 1 [file biology-11-01018-s001.zip › Supplementary Data S2.pdf]
